# Challenges in Optimizing a Prostate Carcinoma Binding Peptide, Identified through the Phage Display Technology

**DOI:** 10.3390/molecules16021559

**Published:** 2011-02-14

**Authors:** Vasileios Askoxylakis, Sabine Zitzmann-Kolbe, Frederic Zoller, Annette Altmann, Annette Markert, Shoaib Rana, Annabell Marr, Walter Mier, Jürgen Debus, Uwe Haberkorn

**Affiliations:** 1 Department of Radiooncology and Radiation Therapy, University of Heidelberg, INF 400, 69120, Heidelberg, Germany; E-Mails: juergen.debus@med.uni-heidelberg.de (J.D.); shoaib.rana@dkfz.de (S.R.); 2 Research Laboratories, Bayer Schering Pharma AG, Berlin, Germany; E-Mail: sabine.zitzmann-kolbe@bayerhealthcare.com (S.Z.-K.); 3 Clinical Cooperation Unit Nuclear Medicine, German Cancer Research Center, INF 260, 69120, Heidelberg, Germany; E-Mails: f.zoller@dkfz-heidelberg.de (F.Z.); a.altmann@dkfz.de (A.A.); a.marr@dkfz.de (A.M.); 4 Department of Nuclear Medicine, University of Heidelberg, INF 400, 69120 Heidelberg, Germany; E-Mails: walter.mier@med.uni-heidelberg.de (W.M.); uwe.haberkorn@med.uni-heidelberg.de (U.H.)

**Keywords:** phage display, peptide, prostate carcinoma, radiolabeling, affinity

## Abstract

The transfer of peptides identified through the phage display technology to clinical applications is difficult. Major drawbacks are the metabolic degradation and label instability. The aim of our work is the optimization of DUP-1, a peptide which was identified by phage display to specifically target human prostate carcinoma. To investigate the influence of chelate conjugation, DOTA was coupled to DUP-1 and labeling was performed with ^111^In. To improve serum stability cyclization of DUP-1 and targeted d-amino acid substitution were carried out. Alanine scanning was performed for identification of the binding site and based on the results peptide fragments were chemically synthesized. The properties of modified ligands were investigated in *in vitro* binding and competition assays. *In vivo* biodistribution studies were carried out in mice, carrying human prostate tumors subcutaneously. DOTA conjugation resulted in different cellular binding kinetics, rapid *in vivo* renal clearance and increased tumor-to-organ ratios. Cyclization and d-amino acid substitution increased the metabolic stability but led to binding affinity decrease. Fragment investigation indicated that the sequence NRAQDY might be significant for target-binding. Our results demonstrate challenges in optimizing peptides, identified through phage display libraries, and show that careful investigation of modified derivatives is necessary in order to improve their characteristics.

## 1. Introduction

Prostate carcinomas are among the most widespread tumors with increasing incidence. In developed countries, prostate cancer represents the most common form of cancer in men, with estimations for 2007 being about 782,600 new cases and 254,000 deaths [1,2]. Early detection is associated with good prognosis and reduced mortality rates. The development within the last years of screening techniques, such as prostate specific antigen (PSA) screening, has led to improvement of disease diagnosis, but PSA screening is also associated with high rates of overdiagnosis and overtreatment [3]. To improve the detection of clinically significant prostate cancer, various imaging tools are used. The most common imaging strategies are transrectal ultrasound (TRUS) and magnetic resonance imaging (MRI). TRUS is widely used for biopsy guidance, while MRI is most commonly performed for determination of the extent of localized disease [4].

Despite improvements in the diagnosis of prostate cancer within the last years, there is still an increased need for the development of good molecular imaging strategies. Such strategies would allow a more accurate visualization of tumor manifestation, resulting not only in more accurate staging but also in improvement of the efficacy of targeted therapeutic modalities. Based on the disease biology, monoclonal antibodies targeting tumor associated antigens represent a promising method for targeted delivery of imaging agents. A prominent example of a prostate cancer associated antigen is the prostate-specific membrane antigen (PSMA), which is expressed in almost all prostate cancer cells, from primary to metastatic disease, and has been used as a target for the development of both radioisotope and chemo-conjugated antibodies [5,6].

However, antibodies often possess disadvantageous properties that do not allow them to be the most favorable format for *in vivo* imaging. Antibodies are slowly cleared from blood circulation, resulting in high background activity for extended periods of time. Their long-circulating half-lives, which is related to the high molecular weight, and the main hepatic clearance lead to lower signal to noise ratios and therefore to reduced contrast in images [7]. Further challenges are their slow extravasation from blood supply and the limited, inhomogeneous penetration of tumor tissue, due to their size [8]. Furthermore, despite progress on the field of protein engineering, leading to production of chimeric or humanized antibodies, their *in vivo* use is often limited by high immunogenicity [9].

As an alternative to antibodies, recent efforts to identify new targeting molecules that could be used for both diagnostic and therapeutic approaches have focused on the use of peptides. Peptides possess pharmacokinetic properties which are advantageous for tumor targeting, such as rapid blood clearance, homogenous tumor penetration and reduced immunogenic potential. Furthermore, peptides are, compared to antibodies, easier and more inexpensive to synthesize [10].

Identification of peptides with specific targeting abilities and tumor affinity is a major challenge in cancer-related peptide research. The phage display technology uses complex random peptide libraries, containing a high number of ligands that are displayed on bacteriophages and represents a very promising tool for identification of specific binding molecules [11]. Phage display libraries have been extensively used for selection of phages expressing peptides on their surface with organ-, protein- or tumor-binding specificity [12,13].

A linear peptide with affinity for prostate carcinoma was identified in our group using phage display techniques. The peptide, DUP-1, was isolated on prostate-specific membrane antigen-negative cells and evaluated both *in vitro* and *in vivo*. Chemical synthesized DUP-1 was tested for binding affinity, specificity, kinetics and internalization. *In vivo* tumor accumulation and organ distribution was evaluated in a nude mouse model, carrying subcutaneously human prostate carcinoma cells. The results of the *in vitro* studies demonstrated a specific binding to prostate carcinoma cells and low binding affinity to non-tumor cells, such as benign prostate or human umbilical vein endothelial cells. Peptide binding was competed with unlabeled DUP-1 and time-dependent internalization into prostate carcinoma cells was demonstrated by confocal microscopy. Organ distribution studies demonstrated a higher accumulation in the tumor than in most of the healthy organs and an increase of radioligand-binding in the prostate tumor up to 300%, compared to normal prostate tissue [14]. These results allow the conclusion that DUP-1 has properties which are very promising for the development of a new diagnostic tracer that specifically targets prostate carcinoma.

However, the transfer of peptide sequences identified through the phage display technology to clinical application and their use for diagnosis or therapy is often difficult. In this respect there are three major aspects that need to be considered and investigated. Firstly, application of a peptide ligand for imaging purposes requires labeling of the molecule. With respect to nuclear medicine applications, labeling with a radionuclide is necessary. The radionuclides that are most used include ^123^I, ^99m^Tc, ^111^In, ^18^F, ^64^Cu and ^68^Ga for diagnostic use or ^90^Y and ^177^Lu for therapeutic use [15]. Various techniques that allow efficient direct or indirect labeling of peptides have been developed within the past three decades. A prominent example of a direct radiolabeling strategy is iodination of the peptide on the side chain of a tyrosine residue [16]. The advantage of direct iodination is that it does not significantly change the size and the conformation of the molecule and, therefore, has a minimized influence on its binding properties. Alternatively, indirect labeling using a chelating moiety covalently bound to the peptide sequence can be performed [17]. The most widely used chelating agents are macrocyclic chelators such as 1,4,7,10-tetraazacyclododecane-1,4,7,10-tetraacetic acid (DOTA) and branched chelators such as diethylenetriaminepentaacetic acid (DTPA). These chelating agents utilize carboxylate and amine groups to form stable complexes with the radioactive metals. In this way, the use of chelators provides an improved labeling stability, which has been demonstrated in various *in vivo* studies [18]. The most prominent example of a chelate conjugated peptide is [DOTA^0^, Tyr^3^]-octreotide (DOTATOC), which is extensively used for diagnosis and therapy of somatostatin-expressing tumors [19,20].

A second major issue in the use of new peptides for clinical applications is their metabolic instability and rapid degradation by serum proteases. Proteolytic instability of a peptide might lead to circulation of short degradation products that do not possess the target affinity of the leader molecule. This could increase the background, resulting in reduced tumor-to-noise ratio. Therefore, peptide stabilization against proteolytic degradation is of high importance, since it might lead to both enhanced tumor accumulation and blood pool decrease. Serum stabilization can be realized by modifications in the ligand sequence. A prominent strategy is peptide cyclization [21]. Evaluation of cyclic analogues of linear, biologically active peptides of somatostatin or melanotropin have demonstrated that cyclic peptides possess the potential to withstand proteolytic degradation, enhance bioavailability and at the same time increase the receptor selectivity [22,23]. Alternative strategies for optimizing the metabolic stability of peptides are targeted modifications of their sequence. Targeted modifications require the identification of the cleavage site in the peptide sequence and include exchange of amino acids with unnatural amino acids, which are not recognized by serum proteases, such as d-amino acids [24], peptide acetylation [25], peptide pegylation [26] or grafting of the binding motif into a stable scaffold structure [27].

Finally, a further major aspect in the optimization of a newly identified peptide ligand prior to its use for targeting purposes is the identification of the binding site in its sequence. This could lead to further reduction of the molecular size and allows targeted modifications, which are often necessary for increased affinity and selectivity, and improved pharmacokinetic properties. A prominent example is the amino acid sequence Arg-Gly-Asp (RGD). RGD has been identified among approximately 2,500 amino acids in the sequence of fibronectin to be responsible for protein binding to integrins [28]. Recognition of the binding tripeptide sequence has led to the development of [(18)F]Galacto-RGD, which is used for imaging of αvβ3 expression in cancer patients [29], as well as the development of cilengitide, which is used in the treatment of glioblastoma [30].

Therefore, aim of the present study was to evaluate the impact of chemical modifications on the sequence of the phage display identified linear dodecapeptide DUP-1 for ligand optimization, with respect of these three important aspects. Since the initial investigation of the peptide was carried out after direct iodination, we firstly investigated the influence of chelate conjugation on the binding and metabolic properties of DUP-1. Thereafter, we focused on optimization of the metabolic stability of DUP-1. Based on the development of peptides which are currently mostly used for clinical applications, like octreotide and RGD, we tested established strategies for improvement of serum stability. These strategies include untargeted modifications, such as peptide cyclization and targeted modifications with amino acid exchange by d-amino acids, after identification of the cleavage site in the peptide sequence. Finally, in order to identify the amino acids that are important for target binding, alanine scanning was carried out and based on the results fragments of DUP-1 were synthesized and tested. In this way, our study aims in identifying molecular modifications, which might lead to optimization of the cellular handling of DUP-1 for targeting prostate cancer. At the same time, our study reveals challenges associated with optimizing phage-display derived peptide molecules prior to their use in clinical studies.

## 2. Results and Discussion

### 2.1. Synthesis and labeling of derivatives and fragments of DUP-1

Various derivatives and fragments of the linear dodecapeptide DUP-1 were synthesized by solid phase peptide synthesis. All peptides synthesized are presented in Table 1. Quality control was performed by HPLC, showing a radiochemical purity >95%. Direct radiolabeling with ^125^I- or ^131^I- was performed on the side group of tyrosine using the chloramine-T method. The specific activity obtained was 90 GBq/µmol for ^125^I- labeling and 110 GBq/µmol for ^131^I- labeling. DOTA-Lys-DUP-1 was labeled with ^111^In. The specific activity obtained was 10 GBq/µmol.

### 2.2. In vitro kinetics, competition and metabolic stability of DOTA-Lys-DUP-1

**Figure 1:**
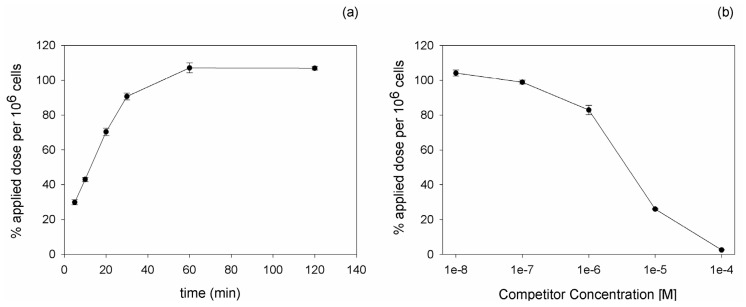
**(a)** *In vitro* cell accumulation of ^111^In-DOTA-Lys-DUP-1 on prostate carcinoma PC-3 cells as a function of time. **(b)** Displacement of bound ^111^In-DOTA-Lys-DUP-1 by the unlabeled DUP-1 peptide at various concentrations on PC-3 cells.

In order to investigate the influence of label on the properties of DUP-1, a chelator was attached to the *C*-terminus of the peptide via a lysine residue and used for ^111^In labeling. After DOTA conjugation and radiolabeling with ^111^In, binding and competition experiments were carried out on PC-3 cells. Kinetic experiments revealed an increasing binding of the peptide with time progression. The maximal radioligand binding was reached after 60 min incubation (Figure 1a). Competition experiments using the unlabeled DUP-1 peptide as competitor at various concentrations demonstrated an increasing inhibition with increasing competitor concentration. At a competitor concentration of 10^−4^ M the recovered lysates showed a binding inhibition of about 98% (p < 0.01), whereas at concentrations below 10^−8^ M the binding value reached the level of uncompeted binding (Figure 1b). The IC_50_ value was calculated as 3.3 µM.

**Figure 2:**
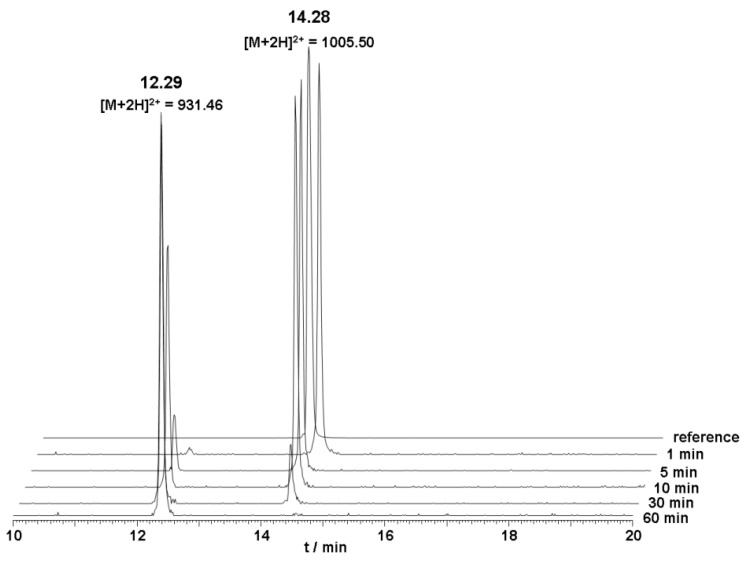
Serum stability of DOTA-Lys-DUP-1. The peptide was incubated in human serum at a concentration of 10^−4^ M.

The metabolic stability of unlabeled DOTA-Lys-DUP-1 was investigated in human serum. The results of the serum stability experiments revealed a serum half life of about 20 min. After 5 min incubation, a small second peak appeared with a retention time of 12.3 min, indicating a first degradation product. Mass analysis revealed that the initial product corresponded to a peptide lacking the first N-terminal amino acid phenylalanine (Δm = 148 Da) (Figure 2).

The conjugation of DOTA had a strong influence on the *in vitro* binding properties of DUP-1. ^125^I-DUP-1 showed a binding of about 12% applied dose per 10^6^ cells, whereas ^111^In-DOTA-Lys-DUP-1 reached 100% applied dose per 10^6^ cells. Furthermore, the DOTA-conjugated peptide showed slower binding kinetics with an increasing binding over time, in contrast to the directly iodinated peptide, which demonstrated a rapid binding with a maximum value after 5 min and a binding decrease thereafter. A possible explanation for this result is an enhanced instability of the label for the iodinated peptide, leading to activity reduction in the cells. Binding reduction over time has been shown in various studies for specific ligands that are directly iodinated through tyrosine residues and internalized into the target cells [31]. This was attributed to the fact that intracellular dehalogenation processes might take place leading to radioactive products that are excreted by the cells [31]. Since DUP-1 has been shown to be quickly internalized into the prostate carcinoma cells [14], intracellular processes, such as dehalogenation might explain the binding decrease for the iodinated molecule, in contrast to the more stable labeled DOTA-conjugated peptide.

However, despite the higher binding values and the improved serum stability of DOTA-Lys-DUP-1 (half life 20 min *vs*. 2 min for DUP-1), DOTA conjugation seems to lead to affinity reduction of the peptide by a factor of approximately 19. The IC_50_ value of ^111^In-DOTA-Lys-DUP-1 is 3.3 µM, while the IC_50_ value of ^125^I-DUP-1 has been shown to be 0.177 µM. This effect can be attributed to changes in the structural conformation and polarity induced by chelator conjugation and insertion of a lysine residue at the C-terminus, phenomenon which has been extensively described in other studies investigating peptides identified by the phage display technology [32].

### 2.3. Biodistribution of radiolabeled ^111^In-DOTA-Lys-DUP-1

**Figure 3:**
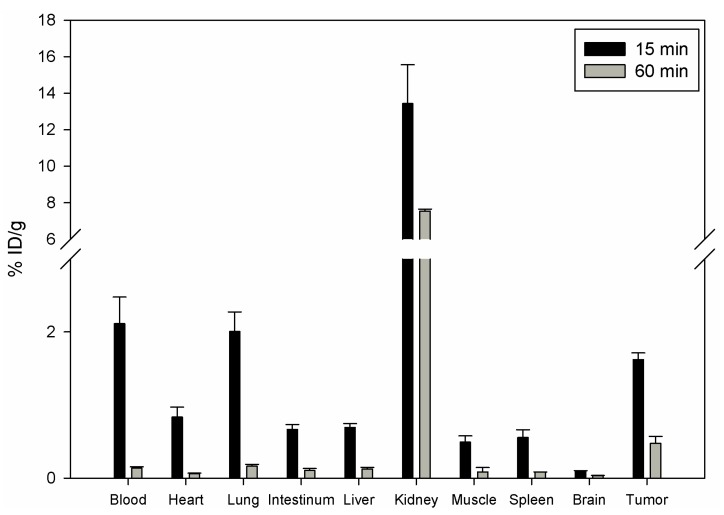
Organ distribution of ^111^In-DOTA-Lys-DUP-1 in Balb/c nu/nu mice carrying PC-3 tumors. Activity concentration (% ID/g) in tumor and control organs is measured after 15 min (n = 3 animals) and 60 min (n = 2 animals) radioligand circulation in the mice. Mean values and standard deviation.

To investigate the influence of DOTA chelate on the *in vivo* distribution of DUP-1, DOTA-Lys-DUP-1 was labeled with ^111^In and injected in the tail vein of nu/nu mice carrying human prostate carcinoma PC-3 cells. The biodistribution showed that DOTA-Lys-DUP-1 reached a level of 1.62% ID/g after 15 min circulation in the blood stream. Binding in heart, spleen, liver, muscle, intestinum and brain was significantly lower (p < 0.05). The highest binding was noticed for the kidney with 13.4%, while blood showed a level of 2.1% after 15 min circulation (Figure 3).

With progression of time activity decrease was noticed for both healthy tissues and tumor (Figure 3). However the decrease for most of the healthy organs, such as blood, heart, lung, spleen, liver, muscle and intestinum was higher than for the tumor, resulting in increase of the tumor-to-organ-ratios (Table 2).

The *in vivo* experiments indicate a faster blood clearance of DOTA-Lys-DUP-1 compared to the native DUP-1 peptide. The level of DOTA-Lys-DUP-1 in blood showed a rapid decrease from 2.1% at 15 min to 0.14% at 60 min post injection. A possible explanation for the rapid clearance is the higher polarity of the DOTA conjugate [32]. Since the *in vitro* data indicated a maximum binding of DOTA-Lys-DUP-1 on PC-3 cells after 60 min incubation, the rapid blood clearance does not allow the peptide to reach its maximum binding capacity *in vivo*, resulting in reduced tumor binding, compared to the iodinated native ligand. This showed a tumor binding of about 7% after 15 min and about 4% after 45 min circulation in mice [14]. The higher clearance of DOTA-Lys-DUP-1 is also demonstrated by the increased radioactivity in the kidneys. The kidney radioactivity at 15 min was about 8% ID/g for the iodinated peptide [14], while it was measured to be 13.5% for the DOTA-conjugated molecule. The fast renal clearance is caused by the hydrophilic chelator DOTA [33].

In spite of the reduced binding capacity of DOTA-Lys-DUP-1, the tumor-to-organ ratios at 15 min were higher for most of the organs compared to the tumor-to-organ ratios of the iodinated molecule [14]. This might be explained by the label instability of the iodinated peptide, resulting in *in vivo* deiodination. *In vivo* deiodination of directly radiolabeled peptides has been described in the literature [34]. Chemical modifications, such as protection of radioiodinated *N*-terminal tyrosine with a *t*-butyloxycarbonyl group, might enhance resistance to peptide deiodination [35].

### 2.4. Improvement of serum stability of DUP-1

**Figure 4:**
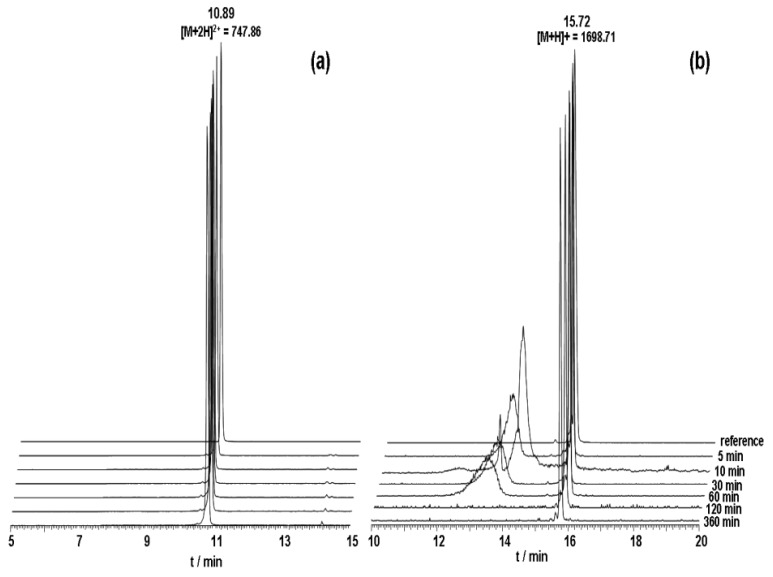
**(a)** Serum stability of dPhe-DUP-1. **(b)** Serum stability of cDUPc. The broad peak at 13.5 min represents serum impurity. Both peptides were incubated in human serum at a concentration of 10^−4^ M.

In order to achieve an improved stability for DUP-1 two different, well established strategies were tested. Firstly, cyclization of the peptide was performed through *N*-terminal and *C*-terminal coupling of cysteines and cyclization via a disulfide bridge (cDUPc). Since metabolic studies have revealed *N*-terminal degradation of DUP-1, targeted modification was applied through replacement of the first *N*-terminal amino acid ^1^Phe by the d-isoform (dPhe-DUP-1). Both strategies were chosen because they are well established for peptide stabilization, while they are chemically easy to perform. The derivatives were labeled with ^125^I and their binding capacity was investigated on PC-3 cells. The results of serum stability experiments demonstrated a significantly increased stability for both derivatives. HPLC analysis showed that full-length peptides were still left after 6 h incubation (Figure 4). Half-life in human serum was >360 min.

**Figure 5:**
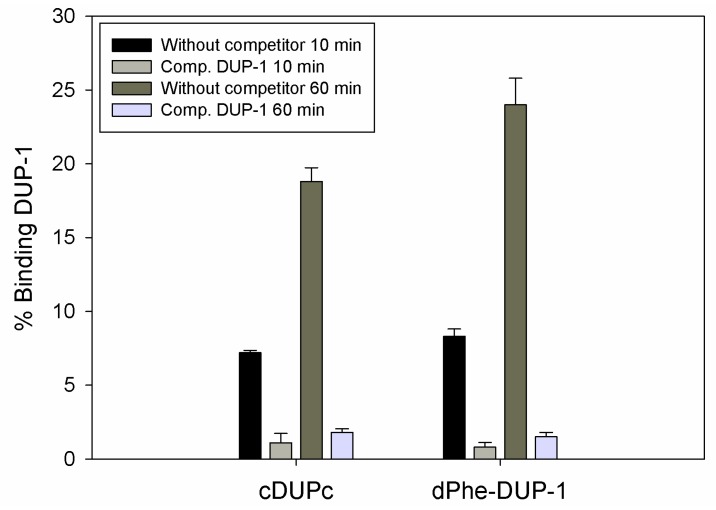
Binding and competition of ^125^I-labeled cDUPc and ^125^I-labeled dPhe-DUP-1 on prostate carcinoma PC-3 cells. The unlabeled DUP-1 peptide was used as competitor at a concentration of 10^−4^ M.

However, binding experiments revealed a strongly reduced binding for both compounds. Binding saturation was achieved for both ligands at 60 min incubation. The cyclic derivative showed a binding of about 7% of the native molecule capacity after 10 min incubation, which increased to about 19% after 60 min incubation. This binding, even though it was lower compared to the native molecule, could be inhibited up to 90% by the unlabeled DUP-1 peptide at a concentration of 10^−4^ M (p < 0.05). For dPhe-DUP-1 binding of about 8.5% of the DUP-1 binding was noticed after 10 min incubation, which increased to about 25% after 60 min incubation. This binding could be inhibited up to 94% by the unlabeled DUP-1 peptide at a concentration of 10^−4^ M (p < 0.05) (Figure 5).

**Figure 6:**
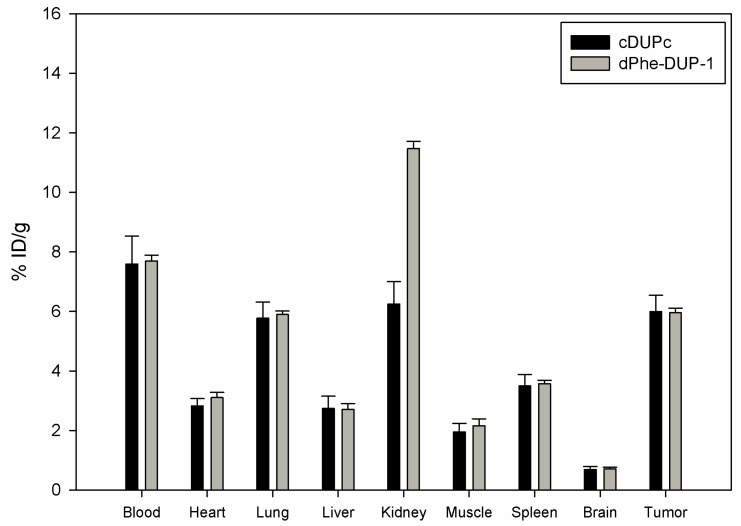
Organ distribution of ^131^I-cDUPc and ^131^I-dPhe-DUP-1 in Balb/c nu/nu mice carrying PC-3 tumors. Activity concentration (% ID/g) in tumor and control organs is measured after 15 min (n = 3 animals) radioligand circulation in the mice. Mean values and standard deviation.

Organ distribution of both peptides was carried out after labeling with ^131^I- in mice carrying PC-3 tumors. The biodistribution experiments showed tumor binding of about 5.8 % ID/g for both cDUPc and dPhe-DUP-1 after 15 min circulation (Figure 6).

Compared to the native molecule, both tumor binding as well as tumor-to-organ ratios were lower (p < 0.05). In particular, *in vivo* binding of cDUPc and dPhe-DUP-1 was about 79% of DUP-1 binding. The *in vitro* experiments showed a binding capacity of about 20% for cDUPc and about 25% for dPhe-DUP-1. The discrepancy between *in vitro* and *in vivo* results is explained by the hypothesis that the enhanced *in vitro* affinity of DUP-1 on PC-3 cells is *in vivo* negatively influenced by its significantly decreased metabolic stability, compared to the two stable derivatives.

d-Amino acids were not only used for stability improvement, but also to prove the specificity of DUP-1 binding. In particular, an enantiomer of DUP-1, consisting of only d-amino acids [d(DUP-1)] was chemically synthesized, labeled with ^125^I- and tested as negative control ligand on PC-3 cells. Indeed, d(DUP-1) showed a significantly decreased binding to a background level of 1-3% of DUP-1 binding (data not shown). The fact that the enantiomer of DUP-1 has a greatly diminished activity is quite promising, since it supports and strengthens the hypothesis of a specific peptide binding.

The results of the investigation of modified derivatives demonstrate the difficulties in stabilizing a newly identified, linear peptide ligand. Both cyclization and exchange of amino acids by unnatural amino acids, such as the d-isoforms might induce conformational changes to the molecule, resulting in reduced binding affinity. This demonstrates a major challenge in optimizing linear peptides identified by phage display libraries. Even if molecules with high binding affinity and specificity are isolated from an array screen and even though chemical strategies to improve serum stability are well established, various modified compounds have to be synthesized and tested, which is both time and cost intensive. To circumvent the mentioned difficulties various modified phage display techniques were developed. A prominent example is the use of peptide libraries based on scaffold structures instead of linear peptides. Due to their restricted conformation scaffold peptides show increased serum stability [27]. Several scaffolds were successfully applied in phage display technology to identify new binders [36]. Affibodies represent an example for a scaffold structure. They consist of three α-helices and are derived from the Z-domain of protein A of *Staphylococcus aureus*. 13 amino acids at the surface of affibodies can be randomized [37]. An affibody molecule, which binds specifically to the extracellular domain of the epidermal growth factor receptor (EGFR) was recently identified [38]. An alternative and elegant method for identification of protein affine peptide ligands with increased stability to enzyme degradation is the mirror-image phage display technology [39]. Mirror image phage display is a technique to identify d-enantiomeric peptides, which are more resistant to proteolytic cleavage [40]. In this approach the target protein is synthesized in the d-amino acid configuration and used to select peptides from a phage display library expressing random l-amino acid peptides. The mirror images of the identified peptides, consisting of metabolic stable d-amino acids, can interact for reasons of symmetry with the natural form of the target protein, consisting of l-amino acids. The technology of mirror-image phage display has been successfully applied for identification of d-peptide antagonists of MDM2, which can be used as ligands for targeted molecular therapy of neoplasms [41].

### 2.5. Identification of the binding site in the sequence of DUP-1

To further characterize the binding site in the sequence of DUP-1 and discriminate which amino acids are responsible for affinity to PC-3 cells, alanine scanning was performed. For alanine scanning, derivatives of DUP-1 were synthesized with exchange of each amino acid by alanine. For radiolabeling of DUP-Ala-9, containing alanine in place of ^9^Tyr, a D-tyrosine was added at the C-terminus. All derivatives were labeled with ^125^I and tested for binding in comparison to native DUP-1. These experiments demonstrated a significant binding decrease for the derivatives DUP-Ala-4, DUP-Ala-7, DUP-Ala-8 and DUP-Ala-9 (p < 0.05). The derivatives DUP-Ala-1, DUP-Ala-2, DUP-Ala-5, DUP-Ala-10, DUP-Ala-11 and DUP-Ala-12 bound almost to the same extent as DUP-1, while the derivative DUP-Ala-3 showed a slight but significant binding increase compared to the native peptide (p < 0.05). The ratio derivative-binding to DUP-1-binding are presented in Table 3.

**Figure 7:**
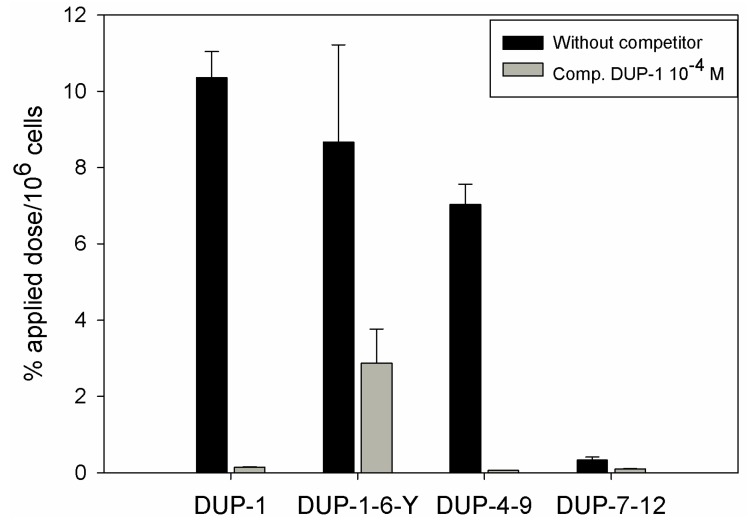
Binding and competition of the DUP-1 peptide fragments DUP-1-6-Y, DUP-4-9 and DUP-7-12 on human prostate carcinoma cells PC-3. The unlabeled DUP-1 peptide was used as competitor at a concentration of 10^−4^ M.

The results of alanine scanning indicate that the amino acids ^4^Asn, ^7^Gln, ^8^Asp and ^9^Tyr might be important for DUP-1 binding on prostate carcinoma cells. To prove this hypothesis, 6-amino acid fragments of the peptide were synthesized, labeled with ^125^I and investigated for binding and competition on human prostate carcinoma PC-3 cells. We chose 6-amino acid fragments because the peptide DUP-4-9, representing the middle part of DUP-1 is the smallest derivative containing all four amino acids that were found by alanine scanning to be important for ligand binding. Comparison after binding saturation of the ^125^I-labeled-fragments DUP-1-6-Y (FRPNRAY), DUP-4-9 (NRAQDY) and DUP-7-12 (QDYNTN) with DUP-1 (FRPNRAQDYNTN) revealed that the fragment DUP-1-6-Y had a binding of about 82% of the leader peptide on PC-3 cells. The peptide DUP-4-9 showed a binding capacity of about 70% of the leader peptide, while the peptide DUP-7-12 showed a reduced binding capacity to the background level (Figure 7). Competition experiments, using the unlabeled DUP-1 peptide at a concentration of 10^−4^ M as competitor revealed a binding inhibition of about 68% for DUP-1-6-Y, and about 99% for DUP-4-9 (Figure 7), which was highly significant (p < 0.01).

These data strengthen the hypothesis that the amino acids ^4^Asn, ^7^Gln, ^8^Asp and ^9^Tyr might be important for peptide binding. In particular, the fragment DUP-4-9, which contains these four amino acids, could be very strongly inhibited by unlabeled DUP-1. Even though the fragment DUP-1-6-Y showed a slight higher binding on PC-3 cells, this binding could not be strongly inhibited by DUP-1, indicating a decrease of specificity and affinity. The fragment DUP-7-12 showed a reduced binding to the background level. This result does not seem at first view to be in concert with the hypothesis generated by alanine scanning, since this fragment includes the amino acids ^7^Gln, ^8^Asp and ^9^Tyr. A possible explanation is that ^4^Asn is an essential amino acid for the binding site, while further *N*-terminal amino acids in the sequence of DUP-1, which did not show a difference within the alanine scanning, might still be important for structural characteristics of the molecule. The absence of those *N*-terminal amino acids might lead to conformational changes, resulting in an affinity decrease.

These results reveal a significant issue in the development of specific binding ligands using the phage display technology. In particular, binding site identification requires the synthesis, labeling and evaluation of a high number of derivatives and fragments of the identified native molecule, which is also time and cost intensive. An efficient method to analyze a large number of derivatives of an identified peptide is the peptide array technology [42,43]. Peptide arrays differ from conventional peptide synthesis in that hundreds upon thousands of peptides are synthesized and presented on a planar surface at a time [44]. After incubation of the target with the peptide array, the interaction between target and peptides can be analyzed. This high-throughput technology allows the rapid identification of peptides which show an increased binding compared to the leader peptide, as well as the detection of peptide motifs, which are essential for binding. Recently peptide arrays were developed to study peptide-whole cell interaction [45]. Nevertheless, the results of peptide arrays should be confirmed by binding assays in following experiments.

### 2.6. In vitro and in vivo evaluation of DUP-4-9

Since binding and competition experiments indicated that DUP-4-9 might be important for target binding, the peptide was further investigated both *in vitro* and *in vivo*. *In vitro* kinetic and competiton studies were performed on PC-3 cells after peptide labeling with ^125^I. These experiments showed binding increase with progression of time and binding saturation after 30 min incubation. Competiton experiments using the unlabeled peptide as competitor revealed an IC_50_ value of 3.5 µM.

**Figure 8:**
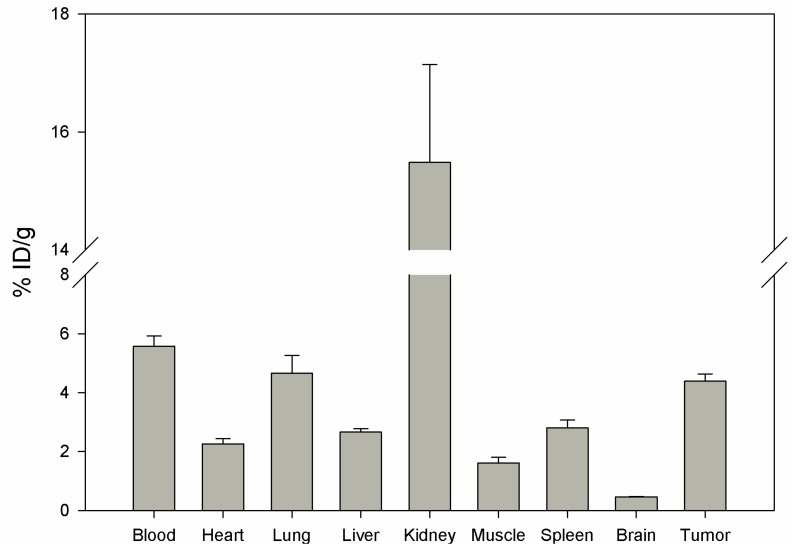
Organ distribution of ^131^I-DUP-4-9 in Balb/c nu/nu mice carrying PC-3 tumors. Activity concentration (% ID/g) in tumor and control organs is measured after 15 min (n = 3 animals) circulation of the radioligand. Mean values and standard deviation.

Biodistribution of DUP-4-9 was investigated in male Balb/c nu/nu mice carrying PC-3 tumors, after labeling with ^131^I. After 15 min circulation the tumor value was about 4.4% ID/g. Binding in heart, spleen, liver, muscle and brain was still significantly lower (p < 0.05). The highest value was measured in the kidneys (15.48%), while radioactivity in blood was calculated to be about 5.5% ID/g (Figure 8).

Although tumor binding was higher than in most organs, calculation of tumor-to-organ ratios showed lower values compared to DUP-1 (data not shown). The results of *in vitro* and *in vivo* studies show a lower binding affinity of DUP-4-9 to target cells, compared to DUP-1. In particular, IC_50_ value and *in vivo* tumor binding were significantly lower (p < 0.05) compared to the leader peptide. These results strengthen the hypothesis that further amino acids, that did not show a difference within the alanine scanning, might still be important for conformational and structural characteristics of the molecule. A truncation library analysis with several derivatives including the NRAQDY sequence can be applied to prove this hypothesis.

## 3. Experimental

### 3.1. Cell lines

The human prostate carcinoma cell line PC-3 (American Type Culture Collection, Manassas, VA, USA) was cultivated at 37 °C in a 5% CO_2_ incubator in RPMI 1640 with Glutamax containing 10% FCS (Invitrogen, Karlsruhe, Germany) and 25 mmol/L HEPES.

### 3.2. Peptides

All peptides were obtained by solid-phase peptide synthesis using Fmoc chemistry. All peptides were analyzed and purified by high-performance liquid chromatography (HPLC) on a P-580 system (Gyncotech) equipped with a variable SPD 6-A ultraviolet detector and a C-R5A integrator (both Shimadzu). The columns used were LiChrosorb RP-select B 5 µm, 250 × 4 mm (Merck, Darmstadt, Germany) and Chromolite® Performance RP-18e, 100 × 3 mm (Merck, Darmstadt, Germany). All analytic runs were performed with a linear gradient of 5%–95% acetonitrile in water at a flow rate of 0.7 mL/min over 30 min, as well as with a linear gradient of 0%–100% acetonitrile + 0.1% TFA at a flow rate of 2 mL/min over 5 min. The mass of the products was determined by mass spectrometry analysis on a matrix-assisted laser desorption ionization time-of-flight mass spectrometer (MALDI-3; Kratos Instruments). Lyophilization was performed on a α_1-2_ lyophilizator (Heraeus-Christ).

### 3.3. Radiolabeling

Direct radiolabeling with ^125^I was performed on the tyrosine moiety using the chloramine-T method. A 10^−3^ M aqueous solution of the respective peptide (5–25 µL, 5–25 nmol) was added to 0.25 M phosphate buffer (25 µL, pH 7.5). A radioactivity amount of 1–10 MBq ^125^I- or ^131^I- was added to this mixture and vortexed for 30 sec. The labeling reaction was quenched by adding 10 µL of a saturated aqueous methionine solution. The radiolabeled peptide was purified by HPLC on a Lichrosorb RP-select B 5 µm, 250 × 4 mm column using a linear solvent gradient system.

For ^111^In-labeling, DOTA was inserted in the sequence of DUP-1 using the preformed lysine conjugate Fmoc-Lys(tris-*t*-BuDOTA)-OH [46]. DOTA-Lys-DUP-1 (H-FRPNRAQDYNTNK(DOTA)-NH_2_) was obtained by solid-phase peptide synthesis using Fmoc-chemistry. Radiolabeling was performed by complexation with ^111^In-chloride. One microliter of a 10^−3^ M solution of DOTA-Lys-DUP-1 in 50% dimethyl sulfoxide in water was mixed with sodium acetate buffer (25 µL, pH 5), and 10 MBq ^111^InCl3 (PerkinElmer, Rodgau) was added. The solution was heated at 95 °C for 25 min. Quality control was performed by HPLC showing a radiochemical purity >95%.

### 3.4. In vitro binding experiments

200,000 cells were seeded in 6-well plates and cultivated for 24 hours. The medium was replaced by 1 mL fresh medium (without FCS). ^125^I-labeled or ^111^In-labeled peptide was added to the cell culture (1–2 × 10^6^ cpm per well) and incubated for various time periods. Thereafter, the cells were washed thrice with 1 mL phosphate buffer saline (PBS) and subsequently lysed with 0.3 mol/L NaOH (0.5 mL). Radioactivity was measured with a γ-counter and calculated as percentage applied dose per 10^6^ cells. For competition experiments co-incubation was performed with the unlabeled peptide at a defined concentration. If BSA or dry milk powder was used as blocking agents, it was added to a final concentration of 1% in medium without FCS.

### 3.5. Organ distribution studies

Male 6-week-old BALB/c nu/nu mice were obtained from Charles River WIGA (Sulzfeld, Germany) and housed in VentiRacks. For inoculation of the tumors, a Matrigel matrix/cell (5 × 10^6^ cells) suspension was injected subcutaneously into the flank of the animals. Tumors were grown up to a size of ~1.0 cm^3^. ^111^In-labeled or ^131^I-labeled peptide was injected via the tail vein into the animals, carrying PC-3 prostate tumors. At defined time points post injection, the mice were sacrificed. Tumor, blood and selected tissues (heart, lung, spleen, liver, kidney, muscle, intestinum and brain) were removed, drained of blood, weighed, and the radioactivity was measured with a γ-counter (LB 951G, Berthold Technologies). The percentage injected dose per gram of tissue (%ID/g) was calculated. All animal experiments were carried out in conformity with the German law for protection of animals and are in compliance with European laws.

### 3.6. Stability experiments

Serum stability experiments were performed with the unlabeled DOTA-Lys-DUP-1, dPhe-DUP-1 and cDUPc peptides. The peptides were incubated in human serum at a concentration of 10^−4^ M at 37 °C. At time points varying from 1 min to 6 hours, aliquots were taken and twice volume of acetonitrile was added, in order to precipitate serum proteins, which were peleted by centrifugation. The supernatant was then analyzed by HPLC-MS (Exactive, Thermo Fisher Scientific Inc.; Agilent 1200 HPLC system) using a linear solvent gradient system (0–100% B, solvent A: 0.1% TFA in water, solvent B: 0.1% TFA in acetonitrile; 200 µL/min, 60 °C; Column: Hypersil Gold C18 column (0.21 × 200 mm, Thermo Fisher Scientific).

### 3.7. Data analysis and statistics

Statistical analysis and comparisons between groups were performed using the student *t* test on the SIGMASTAT program (Jandel Scientific). A p value of 0.05 or less was considered statistically significant.

## 4. Conclusions

In order to optimize the affinity and metabolic properties of a new prostate carcinoma binding peptide identified by phage display technology we investigated the impact of modifications in the amino acid sequence. We focused on three major aspects that need to be evaluated prior to the use of a ligand for targeting or imaging purposes: investigation of the influence of chelate conjugation for radiolabeling, improvement of the metabolic stability and identification of the binding site in the sequence of the peptide. DOTA conjugation resulted in different cellular binding kinetics, rapid *in vivo* renal clearance and increased tumor-to-organ ratios. Cyclization and targeted d-amino acid substitution increased the metabolic stability but led to a significant binding affinity decrease. Alanine scanning indicated the amino acids that are important for peptide binding, while fragment analysis revealed that the sequence NRAQDY might be significant for prostate carcinoma targeting.

Our study reveals challenges in the optimization of a linear peptide after identification using the phage display technology. Our results demonstrate that the tumor-targeting properties of phage display identified peptides can be strongly modulated through chemical modifications of the ligand. This effect can be attributed to structural and conformational changes induced by the applied modifications. Although there are established strategies for metabolic stability improvement, careful probing of the pharmacologic properties of various modified derivatives is necessary, which is time and cost intensive. Identification of the binding site in the amino acid sequence of a phage display isolated peptide is of high importance, since it could allow targeted alterations, aiming in stabilized, specific binding ligands with enhanced target affinity.

## Figures and Tables

**Table 1:** Analytical data of synthesized peptides.

Peptide name	Sequence	Retention time [min]	Purity [%]	Mass (g/mol) detec. [M+2H]^2+^	Mass (g/mol) calc. [M+2H]^2+^
DUP-1	FRPNRAQDYNTN	1,74	>99	747.8627	747.8634
DOTA-Lys-DUP-1	FRPNRAQDYNTN-K-DOTA	1.694	>99	1005.001	1005.001
cDUPc	C-FRPNRAQDYNTN-C	1.819	>99	849.8644	849.8648
dPhe-DUP-1	fRPNRAQDYNTN	1.75	>99	747.8627	747.8634
d(DUP-1)	frpnraqdyntn	1.698	>95	747.8643	747.8634
DUP-1-6-Y	FRPNRAY	1.811	>95	462.2453	462.2459
DUP-4-9	NRAQDY	1.511	>99	383.1847	383.1855
DUP-7-12	QDYNTN	1.466	>95	377.1610	377.1617
DUP-Ala-1	ARPNRAQDYNTN	1.527	>99	709.8494	709.8478
DUP-Ala-2	FAPNRAQDYNTN	1.715	>99	705.3322	705.3314
DUP-Ala-3	FRANRAQDYNTN	1.676	>95	734.8568	734.8556
DUP-Ala-4	FRPARAQDYNTN	1.718	>99	726.3619	726.3605
DUP-Ala-5	FRPNAAQDYNTN	1.759	>95	705.3328	705.3314
DUP-Ala-7	FRPNRAADYNTN	1.720	>95	719.3544	719.3527
DUP-Ala-8	FRPNRAQAYNTN	1.733	>95	725.8699	725.8685
DUP-Ala-9	FRPNRAQDANTNy	1.726	>95	783.3823	783.3820
DUP-Ala-10	FRPNRAQDYATN	1.748	>95	726.3613	726.3605
DUP-Ala-11	FRPNRAQDYNAN	1.694	>95	732.8583	732.8582
DUP-Ala-12	FRPNRAQDYNTA	1.724	>95	726.3612	726.3605

Column: Chromolite® Performance RP-18e, 100 × 3 mm; Gradient: 0%–100% MeCN + 0.1% TFA in 5 min; Flow: 2 mL/min.

**Table 2:** Tumor-to-organ ratios of ^111^In-DOTA-Lys-DUP-1 in mice carrying PC-3 tumors.

Tumor-to-organ ratio	15 min	60 min
Blood	0.790 ± 0.198	3.454 ± 0.261
Heart	1.992 ± 0.459	7.788 ± 0.578
Lung	0.823 ± 0.167	2.897 ± 0.142
Intestine	2.456 ± 0.391	4.790 ± 2.128
Liver	2.348 ± 0.196	3.807 ± 0.070
Kidney	0.124 ± 0.028	0.063 ± 0.012
Muscle	3.392 ± 0.833	8.047 ± 6.848
Spleen	3.015 ± 0.793	5.705 ± 1.086
Brain	18.59 ± 4.047	14.84 ± 4.734
Tumor	1.0	1.0

**Table 3:** Alanine scanning of DUP-1. Ratios binding-derivative to binding DUP-1.

Peptide	Sequence	Ratio binding-derivative to binding-DUP-1
DUP-1	FRPNRAQDYNTN	1
DUP-Ala-1	ARPNRAQDYNTN	0.986
DUP-Ala-2	FAPNRAQDYNTN	0.967
DUP-Ala-3	FRANRAQDYNTN	1.222
DUP-Ala-4	FRPARAQDYNTN	0.794
DUP-Ala-5	FRPNAAQDYNTN	1.035
DUP-Ala-7	FRPNRAADYNTN	0.814
DUP-Ala-8	FRPNRAQAYNTN	0.689
DUP-Ala-9	FRPNRAQDANTNy	0.581
DUP-Ala-10	FRPNRAQDYATN	0.964
DUP-Ala-11	FRPNRAQDYNAN	0.969
DUP-Ala-12	FRPNRAQDYNTA	0.963
